# Supplementary material for: Conversion of the Native N-Terminal Domain of TDP-43 into a Monomeric Alternative Fold with Lower Aggregation Propensity
Source: Molecules. 2022 Jul 5;27(13):4309. doi: 10.3390/molecules27134309 (PMC9268139; doi:10.3390/molecules27134309)
Supplement: Supplementary file 1 [file molecules-27-04309-s001.zip › molecules-1768149-supplementary.pdf]

Supporting Information for

Conversion of the native N-terminal domain of TDP-43 into a monomeric alternative fold with lower aggregation propensity

Matteo Moretti[1,*], Isabella Marzi[1,*], Cristina Cantarutti[2], Mirella Vivoli Vega[3], Walter Mandaliti[2], Maria Chiara Mimmi[4], Francesco Bemporad[1], Alessandra Corazza[2,5], Fabrizio Chiti[1,^]

[1] *Department of Experimental and Clinical Biomedical Sciences "Mario Serio", University of Florence, FI 50134, Italy.*

[2] *Department of medical area, University of Udine, UD 33100, Italy.*

[3] *School of Biochemistry, University of Bristol, BS8 1TD Bristol, United Kingdom.*

[4] *Department of Molecular Medicine, University of Pavia, PV 27100, Italy.*

[5] *INBB, Viale Medaglie d'Oro 305, Roma 00136, Italy.*

[*] These authors contributed equally to the work

[^] Corresponding author: Fabrizio Chiti; Viale Giovanni Battista Morgagni,50; +39 055 275 1220; fabrizio.chiti@unifi.it

Table S1. Comparison between secondary structures of native TDP-43 NTD and the alternative conformation. Comparison performed according to CSI and Talos+ analysis and NH involvement into H bonds as inferred from temperature coefficients. H stands for α-helix, E for extended β-strand and x indicates uncertainty.

Secondary structure comparison								
	Native (PDB 5x4f)	Native (PDB 6b1g)	Form A (3% SB31-10)			Form B (3% SB31-10)		
Residue			CSI	Talos+	H bond	CSI	Talos+	H bond
M1			H		yes	H		yes
S2			H	H	yes	H	H	yes
E3			H	H	yes	H	H	yes
Y4		E		H	x		H	x
I5	E	E	E		yes	E		yes
R6	E	E			yes			yes
V7	E	E			yes			yes
T8		E	E		x	E		x
E9					yes			yes
D10					no			no
E11					no			no
N12					no			no
D13					no			no
E14					no			no
P15	-		-	-	-	-	-	-
I16	E	E	E		no	E		no
E17	E	E	E	E	no	E	E	no
I18	E	E	E		no	E		no
P19	-		-	-	-	-	-	-
S20					x			x
E21			-	-	-	-	-	-
D22					no			no
D23					no			no
G24			H		x			x
T25	E	E			yes			no
V26	E	E	H	H	no			yes
L27	E	E	H	H	x			no
L28	H	H	H	H	yes	H	H	no
S29	H	H	H	H	yes	H	H	yes
T30	H	H	H	H	x	H	H	x
V31	H	H	H	H	no	H	H	no
T32	H	H	H	H	no	H	H	no
A33	H	H	H	H	yes	H	H	yes
Q34		H			yes			yes
F35		H			x			x
P36	-		-	-	-	-	-	-
G37					x			x
A38					no			no
C39					yes			yes
G40	E				yes			yes
L41	E	E			no			no
R42	E	E			no			no
Y43	E	E			no			no

R44	E	E	-	-	-	-	-	-
N45			-	-	-	-	-	-
P46	-		-	-	-	-	-	-
V47					x			x
S48					no			no
Q49					no			no
C50			-	-	-	-	-	-
M51		E	-	-	-	-	-	-
R52		E			yes			yes
G53		E			no			no
V54			E		no	E		no
R55	E	E	-	-	-	-	-	-
L56	E	E	-	-	-	-	-	-
V57	E	E	E		x	E		x
E58					yes			yes
G59					no			no
I60	E	E			no			no
L61	E	E			no			no
H62	E	E			no			no
A63					no			no
P64	-		-	-	-	-	-	-
D65					no			no
A66					no			no
G67					yes			yes
W68			H		no	H		no
G69			H		no	H		no
N70			H	H	x	H	H	x
L71				H	yes		H	yes
V72	E			H	yes		H	yes
Y73	E	E		H	yes		H	yes
V74	E	E			yes			yes
V75	E	E			yes			yes
N76	E				yes			yes
Y77					yes			yes

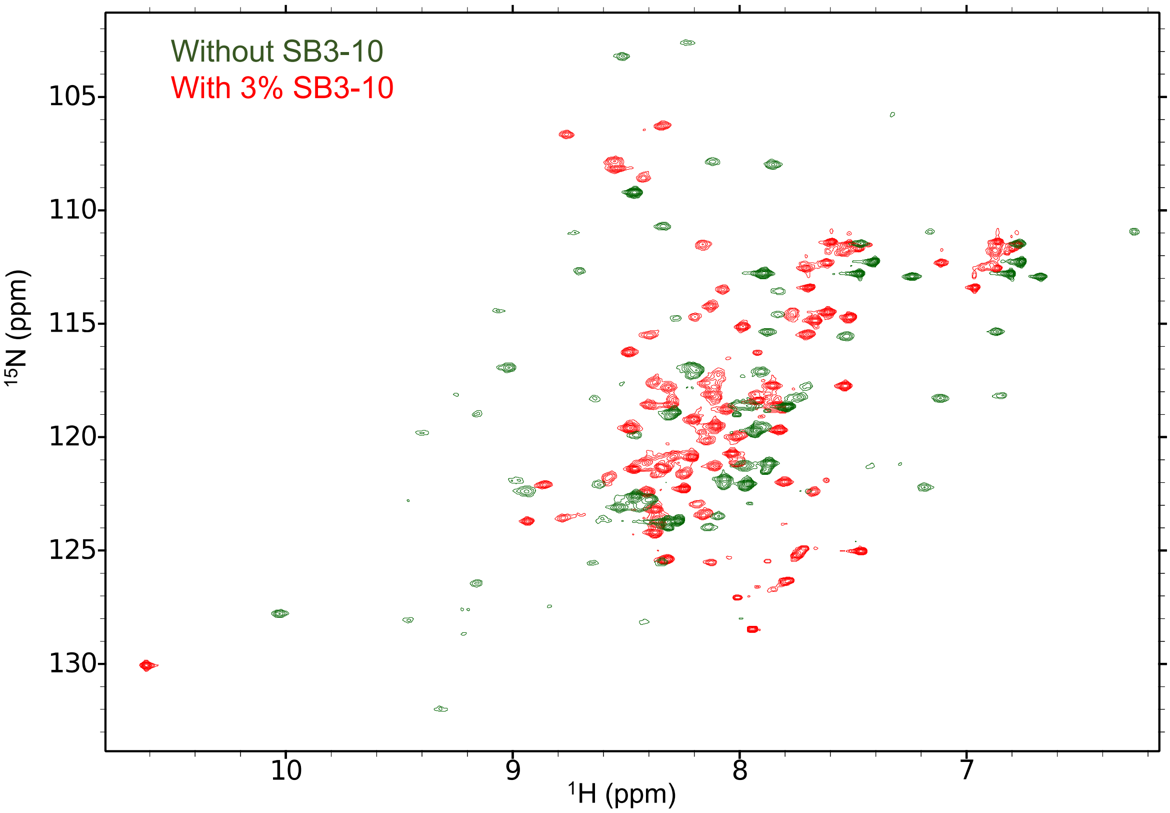

Figure S1. Change of ^{1}H-^{15}N HSQC spectrum upon addition of 3% SB3-10. Overlay of ^{1}H-^{15}N HSQC spectra of TDP-43 NTD recorded at 500 MHz and 25 °C in absence (green) and presence (red) of 3% (w/v) SB3-10. The superimposition of the two spectra clearly shows the reduced spectral dispersion in the presence of 3% SB3-10.

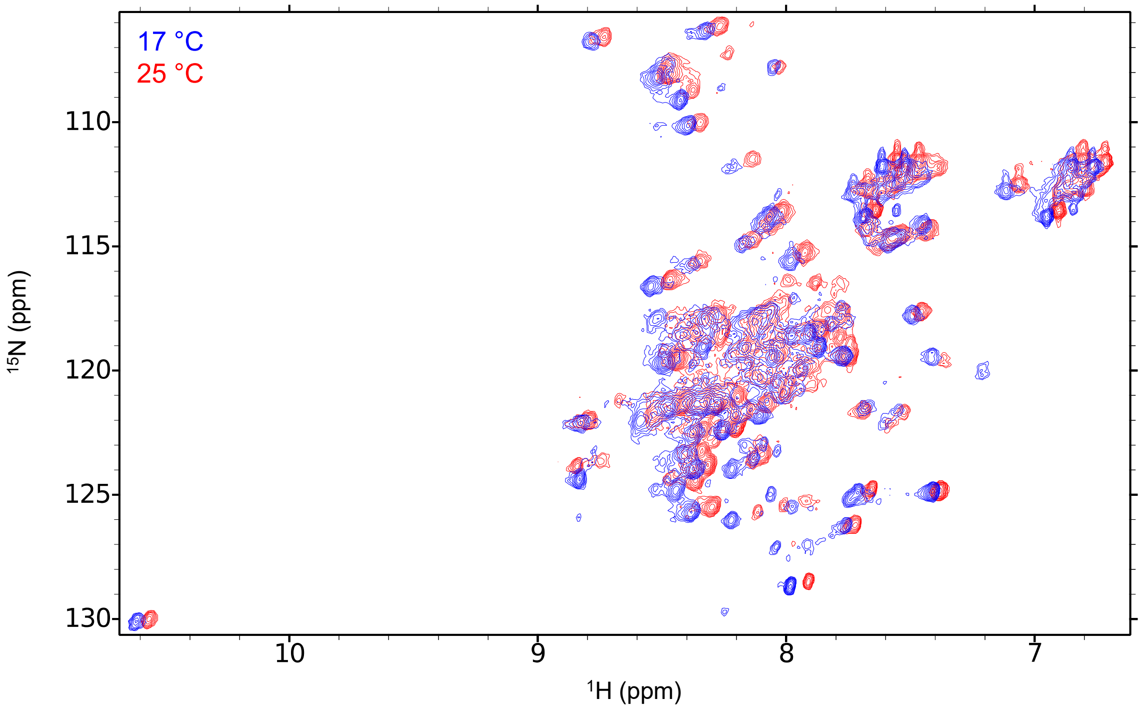

Figure S2. Change of ^{1}H-^{15}N HSQC spectrum upon temperature decrease. Overlay of ^{1}H-^{15}N HSQC spectra of TDP-43 NTD recorded at 700 MHz at 25 °C (red) and 17 °C (blue) in the presence of 3% (w/v) SB3-10. The superimposition of the two spectra shows the increase in the number of backbone amide peaks at lower temperature.

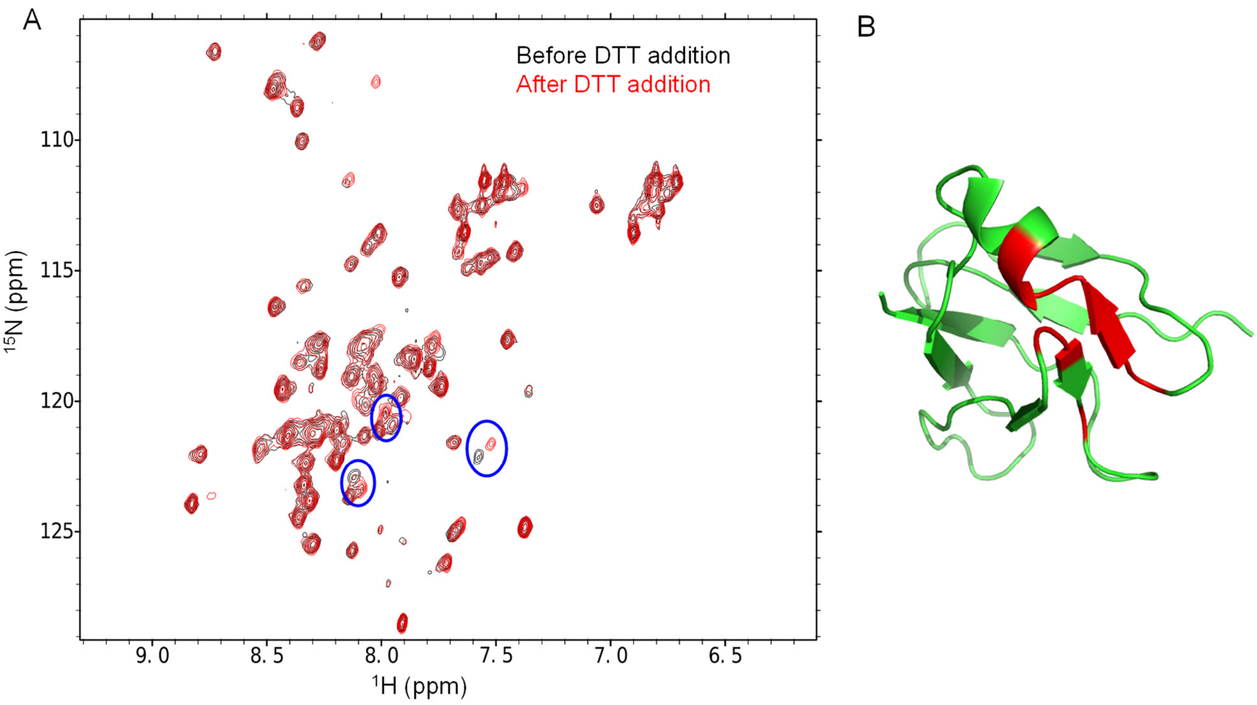

Figure S3. Change of ^{1}H-^{15}N HSQC spectrum upon DTT addition (**A**) Superimposition of ^{1}H-^{15}N HSQC spectra of the TDP-43 NTD alternative conformation recorded at 700 MHz and 25 °C before (black) and after (red) the addition of freshly prepared DTT. The blue circles highlight peaks where the second form is highly decreased after DTT addition. (**B**) Native TDP-43 NTD protein cartoon (pdb 5x4f) showing the residues presenting double forms in the HSQC spectrum that are not affected by DTT addition coloured in red.

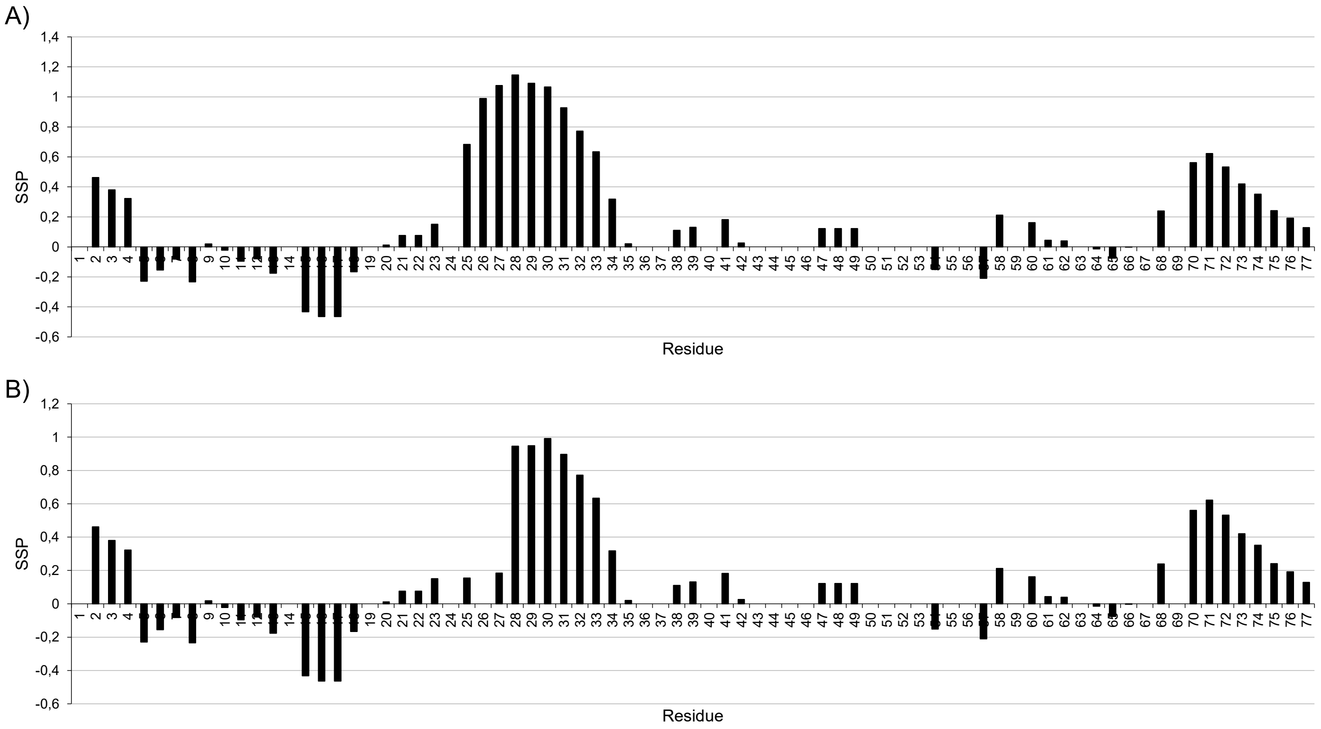

Figure S4. SSP analysis of alternative TDP-43 NTD conformation for both form A (A) and form B (B). SSP indicates the tendency to adopt secondary structure (SSP > 0 corresponds to α-helix, SSP < 0 corresponds to β-strand). A SSP score of ± 1 corresponds to fully formed secondary structure, while an intermediate SSP value indicate a propensity for those residues to adopt the corresponding secondary structure.

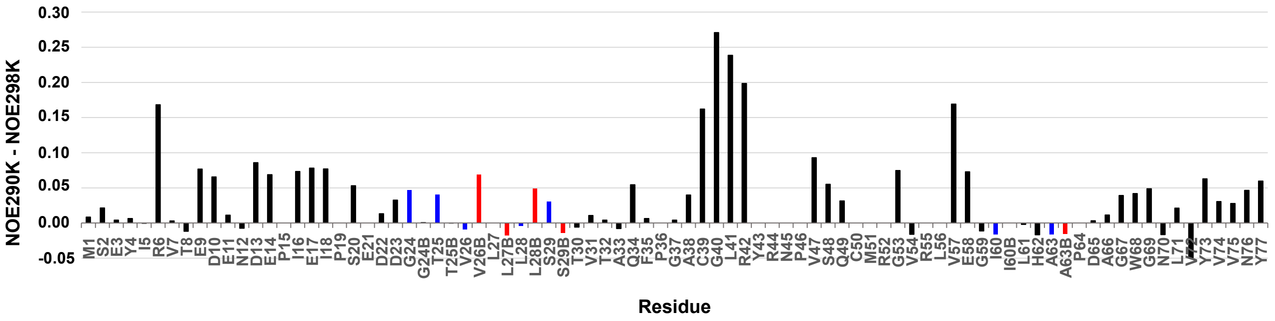

Figure S5. Difference between NOE values found at different temperature values. The differences were obtained at 17 °C (290 K) and 25 °C (298 K) for the TDP-43 NTD alternative form in 3% (w/v) SB3-10. The black, blue and red bars correspond to both forms, form A and form B, respectively.

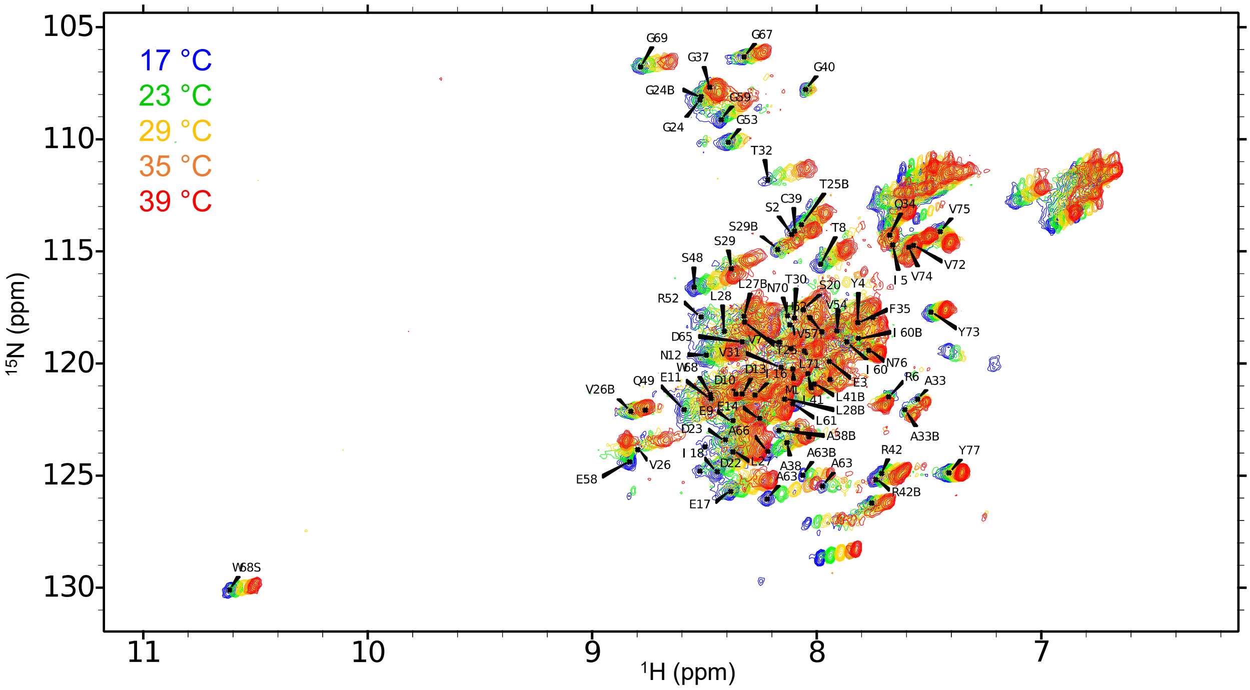

Figure S6. Overlay of ^{1}H-^{15}N HSQC spectra of TDP-43 NTD. Spectra are in 3% (w/v) SB3-10 recorded at 700 MHz at different temperatures: 17 °C (blue), 23 °C (green), 29 °C (yellow), 35 °C (orange) and 39 °C (red).

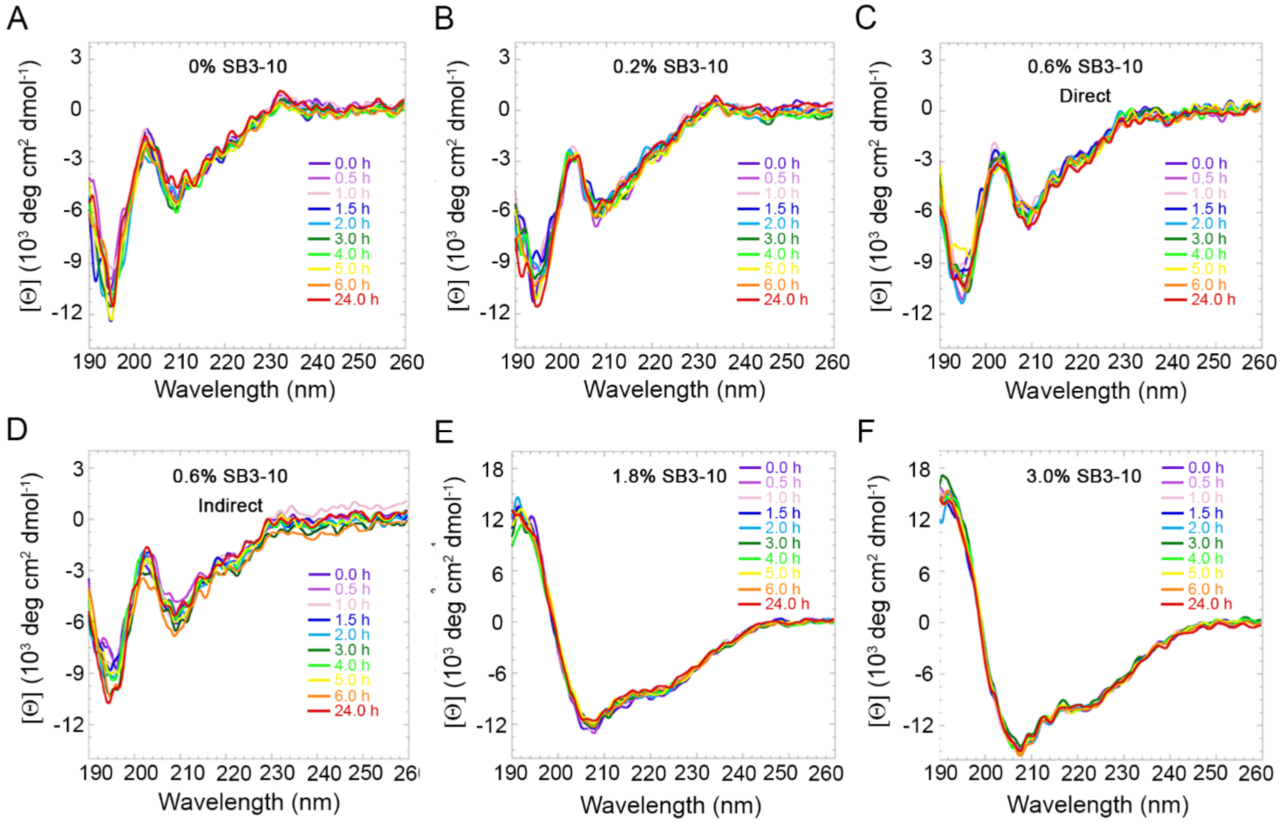

Figure S7. Aggregation kinetics of TDP-43 NTD at different SB3-10 percent concentrations monitored with far-UV CD. Far-UV CD spectra of TDP-43 NTD (0.5 mg/ml, 45 µM) in 5 mM sodium phosphate buffer, 50 mM NaCl, 1 mM DTT, pH 7.4, 25 °C, in the presence of 0.0% (w/v) SB3-10 (**A**), 0.2% (w/v) SB3-10 (B), 0.6% (w/v) SB3-10 (**C,D**), 1.8% (w/v) SB3-10 (**E**), 3.0% (w/v) SB3-10 (**F**), at the indicated time points. In panel **D** TDP-43 NTD was pre-incubated in 1.8% (w/v) SB3-10 for 1 h and then diluted to 0.6% (w/v) SB3-10 at the same final conditions as in panel **C**.

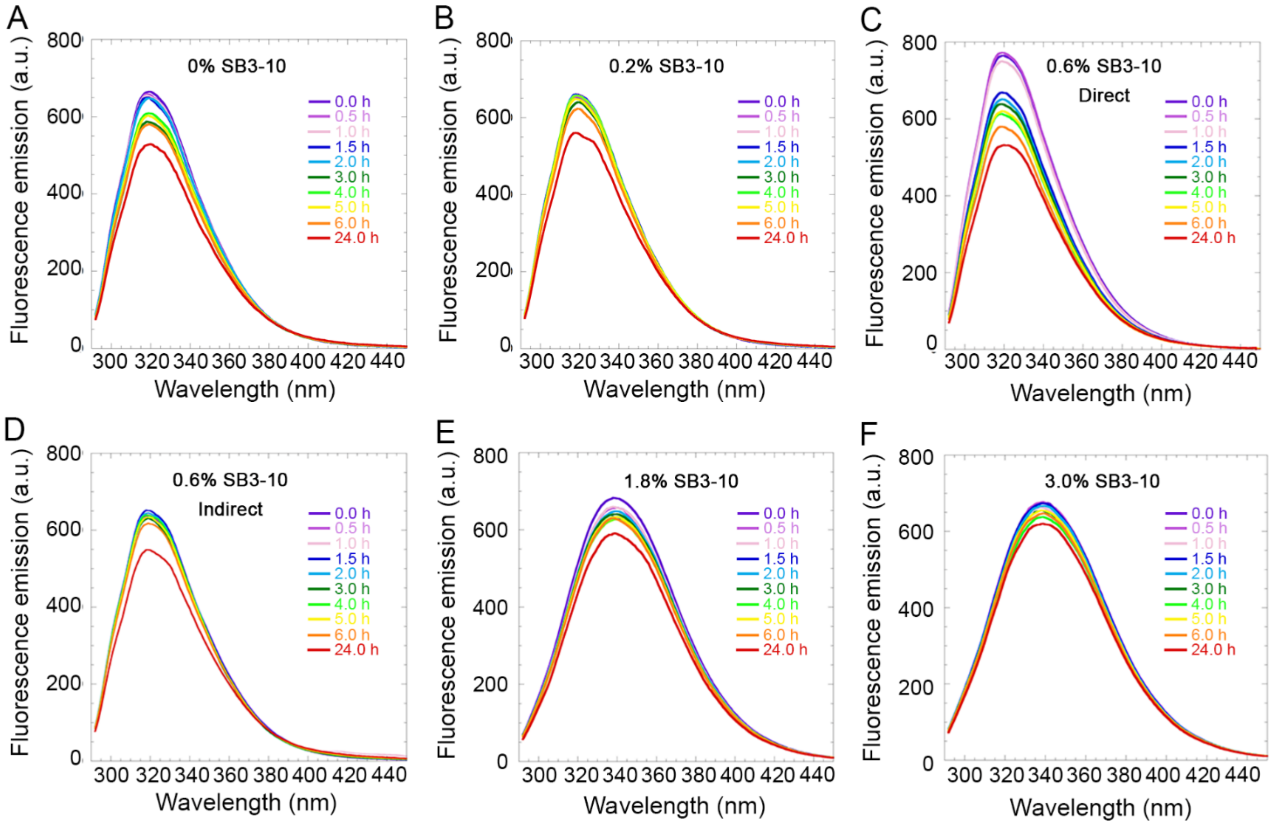

Figure S8. Aggregation kinetics of TDP-43 NTD at different SB3-10 percent concentrations monitored with intrinsic fluorescence. Intrinsic Fluorescence spectra of TDP-43 NTD (0.5 mg/ml, 45 μM) in 5 mM sodium phosphate buffer, 50 mM NaCl, 1 mM DTT, pH 7.4, 25 °C, in the presence of 0.0% (w/v) SB3-10 (**A**), 0.2% (w/v) SB3-10 (**B**), 0.6% (w/v) SB3-10 (**C,D**), 1.8% (w/v) SB3-10 (**E**), 3.0% (w/v) SB3-10 (**F**), at the indicated time points. In panel **D** TDP-43 NTD was pre-incubated in 1.8% (w/v) SB3-10 for 1 h and then diluted to 0.6% (w/v) SB3-10 at the same final conditions as in panel **C**.
